# Accuracy of ^177^Lu activity quantification in SPECT imaging: a phantom study

**DOI:** 10.1186/s40658-016-0170-3

**Published:** 2017-01-07

**Authors:** Carlos F. Uribe, Pedro L. Esquinas, Jesse Tanguay, Marjorie Gonzalez, Emilie Gaudin, Jean-Mathieu Beauregard, Anna Celler

**Affiliations:** 1 Medical Imaging Research Group, Department of Radiology, University of British Columbia, Vancouver, British Colombia Canada; 2 Department of Physics and Astronomy, University of British Columbia, Vancouver, British Colombia Canada; 3 Department of Molecular Oncology, BC Cancer Research Centre, Vancouver, British Colombia Canada; 4 Vancouver Coastal Health Authority, Vancouver, British Colombia Canada; 5 Department of Physics, Engineering Physics and Optics, Université Laval, Quebec City, Quebec Canada; 6 Department of Medical Imaging, CHU de Quebec–Université Laval, Quebec City, Quebec Canada; 7 Department of Radiology and Nuclear Medicine, Université Laval, Quebec City, Quebec Canada

**Keywords:** ^177^Lu, Quantification, Dosimetry, TEW, APDI, Scatter correction

## Abstract

**Background:**

The aim of the study is to assess accuracy of activity quantification of ^177^Lu studies performed according to recommendations provided by the committee on Medical Internal Radiation Dose (MIRD) pamphlets 23 and 26. The performances of two scatter correction and three segmentation methods were compared. Additionally, the accuracy of tomographic and planar methods for determination of the camera normalization factor (CNF) was evaluated.

Eight phantoms containing inserts of different sizes and shapes placed in air, water, and radioactive background were scanned using a Siemens SymbiaT SPECT/CT camera. Planar and tomographic scans with ^177^Lu sources were used to measure CNF. Images were reconstructed with our SPEQToR software using resolution recovery, attenuation, and two scatter correction methods (analytical photon distribution interpolated (APDI) and triple energy window (TEW)). Segmentation was performed using a fixed threshold method for both air and cold water scans. For hot water experiments three segmentation methods were compared as folows: a 40% fixed threshold, segmentation based on CT images, and our iterative adaptive dual thresholding (IADT). Quantification error, defined as the percent difference between experimental and true activities, was evaluated.

**Results:**

Quantification error for scans in air was better for TEW scatter correction (<6%) than for APDI (<11%). This trend was reversed for scans in water (<10% for APDI and <14% for TEW). For hot water, the best results (<18% for small objects and <5% for objects >100 ml) were obtained when APDI and IADT were used for scatter correction and segmentation, respectively. Additionally, we showed that planar acquisitions with scatter correction and tomographic scans provide similar CNF values. This is an important finding because planar acquisitions are easier to perform than tomographic scans. TEW and APDI resulted in similar quantification errors with APDI showing a small advantage for objects placed in medium with non-uniform density.

**Conclusions:**

Following the MIRD recommendations for data acquisition and reconstruction resulted in accurate activity quantification (errors <5% for large objects). However, techniques for better organ/tumor segmentation must still be developed.

**Electronic supplementary material:**

The online version of this article (doi:10.1186/s40658-016-0170-3) contains supplementary material, which is available to authorized users.

## Background

Targeted radionuclide therapy (TRT) uses pharmaceuticals labeled with radioisotopes which emit particles (β’s or α’s) to deliver dose directly to tumors, while avoiding irradiating healthy tissues. This approach has been used in management of a number of oncological and other disorders [1, 2]. In particular, TRT has been shown to produce very encouraging results in treatment of neuroendocrine tumors (NETs) using ^177^Lu labeled peptide analogs [3]. Unfortunately, treatment outcomes vary greatly between patients and cure remains rare. There is growing evidence that this lack of consistent response may be due to the “one-size-fits-all” treatment approach, where all patients are injected with the same, relatively low activity of approximately 7400 MBq/cycle [4]. However, radiotracer uptake in tumors and healthy tissue varies greatly between patients, which results in a big group of under-treated individuals [5, 6]. It is generally believed that treatment plans using injections based on individualized dose assessments could significantly improve TRT outcomes; therefore, they should become routine, as is already the case in external beam therapies [7, 8].

This opinion prompted the recent publication of a series of committee on Medical Internal Radiation Dose (MIRD) pamphlets specifying guidelines for SPECT-based activity quantification which is necessary for personalized, image-based dosimetry for TRT. These publications clearly identify the sequence of procedures which have to be performed and corrections which have to be applied to generate quantitative images of activity distribution in the patient body and to obtain information about how this activity changes over time. The general recommendations are outlined in MIRD pamphlet 23 [9], while MIRD pamphlet 26 [10] focuses specifically on studies performed with ^177^Lu.

However, these documents do not provide information about the accuracy of activity quantification that can be achieved when following these guidelines. Additionally, in some cases, the recommended procedures or corrections are not uniquely defined and the final selection of the method is left to the user. Consequently, the decision regarding which method should be used may be difficult, especially if the accuracies of different approaches are not uniquely characterized.

Previous studies investigating the accuracy of ^177^Lu quantification used a number of different phantom configurations, and different data acquisition and processing methods. For example, Beauregard et al. [11] measured activity of 175 ml cylinders placed in a large cylindrical phantom and reported 6.6 ± 3.5% average errors. They used camera manufacturer’s image reconstruction software with attenuation and dual energy window (DEW) [12] scatter corrections, while contributions from scattered high-energy photons were ignored. Our group's previous ^177^Lu quantification studies [13], based on the 113 keV photopeak using low-energy high-resolution (LEHR) collimator and the analytical photon distribution interpolated (APDI) scatter correction method [14, 15], resulted in 2% accuracy error for a 70 ml container scanned in air and in water (without background activity). In parallel, seven scatter correction methods and different collimators were evaluated by de Nijs et al. [16] using phantom containing hot spheres in warm background with concentration ratio close to 13:1. Although triple energy window (TEW) [17] was found to not be suitable when imaging the 113 keV photopeak, for the 208 keV peak TEW, or even DEW method, resulted in activity estimation errors close to 10% for the largest 37 ml sphere. Sanders et al. [18] scanned a cylinder uniformly filled with activity containing small spheres (0.5–16 ml) and used TEW scatter correction. The resulting quantification errors were about 20% for the largest sphere. Most recently, Hippeläinen et al. [19] tested the accuracy of quantification using spheres filled with ^177^Lu placed inside a torso phantom. The ratio of spheres to background activity concentrations was very high (30:1). The best quantification was achieved when attenuation, collimator response, and Monte Carlo-based scatter corrections were implemented in the reconstruction algorithm, and 15% error was reported for the largest sphere.

Once quantitatively accurate images are reconstructed, the counts in each voxel must be translated into activity values [9, 10]. This is done using experimentally determined camera normalization factor (CNF), and both planar and tomographic methods have been proposed for this purpose. While planar acquisitions are easier and faster to perform than tomographic scans, it is believed that they can only be used when highly accurate scatter and attenuation corrections are included in image reconstructions. Tomographic acquisitions are said to better approximate scatter and attenuation in patients resulting in better compensation for possible errors in the quantitative reconstructions [9]. We decided to verify these claims in a challenging case of ^177^Lu.

Finally, once the images of quantitative activity distributions are available, to perform organ dosimetry (for example, following OLINDA protocol [20]), the exact information about organ of interest activity and its mass are required. Considering this task, we compared the accuracy of activity quantification for objects scanned in hot background. Three different segmentation approaches were evaluated: (i) a 40% fixed threshold method which is commonly used in clinical studies [21], (ii) segmentation based on true organ volume obtained directly from CT images, and (iii) our iterative adaptive dual thresholding (IADT) method [22].

To summarize, using a large series of phantom experiments, we investigated the following four questions:

1. What accuracy of ^177^Lu activity quantification can be achieved in phantom experiments performed following recommendations of the MIRD pamphlet 26?
2. What is the accuracy of quantification for the two scatter correction methods:(i) the fast and easy, but approximate TEW method [17], and (ii) a rigorous, but computationally intensive APDI [14, 15] method.
3. What is the best method to measure the camera normalization factor (CNF), and what are the uncertainties associated with each of these methods?
4. How is the accuracy of activity quantification in an organ affected by the employed segmentation method?

## Methods

To answer the questions listed in the previous section, we performed a series of experiments using a wide range of phantom geometries and different attenuation and scatter conditions.

1. Phantoms with hot inserts placed in air. The purpose of scanning hot objects of different shapes and sizes placed in air was to evaluate the accuracy of our reconstructions in situations with relatively small amount of attenuation and scatter.
2. Phantoms with hot inserts placed in water. Hot objects (with activity added) placed in cylinders filled with cold water (no activity added) allowed us to compare the performance of the two scatter correction methods. In these experiments phantoms with uniform (Jaszczak cylinder) and non-uniform (thorax phantom) density distributions were used.
3. Phantoms with hot inserts in warm water (containing activity). The purpose of experiments with activity in the background was to test the performance of the three different segmentation approaches in determination of the activities and the volumes of objects of different sizes. The configuration with hot objects in warm background best approximates the conditions present in patient studies.
4. Phantoms for CNF and IADT calibrations. The objective of these additional experiments (performed separately from the series discussed above) was to (a) measure the camera normalization factor using images from tomographic acquisitions of objects with different levels of activity, and (b) determine thresholding curves for the IADT segmentation method.

The experimental details and the procedures are explained in the following sections.

### Data acquisitions

All scans were performed using a SymbiaT (Siemens Medical, Germany) SPECT/CT camera with a medium-energy low-penetration (MELP) collimator. For each tomographic scan 90 projections were acquired with a non-circular orbit using a 128 x 128 matrix and three energy windows: the 208 keV photopeak window (PW), lower scatter window (LSW), and upper scatter window (USW) (see Table 1).

**Table 1:** Energy window used in the ^177^Lu phantom experiments

Name	Lower limit [keV]	Center [keV]	Upper limit [keV]
LSW	153.0	170.0	187.0
PW	187.2	208.0	228.8
USW	229.5	255.0	280.2

### Phantom experiments

**Fig. 1:**
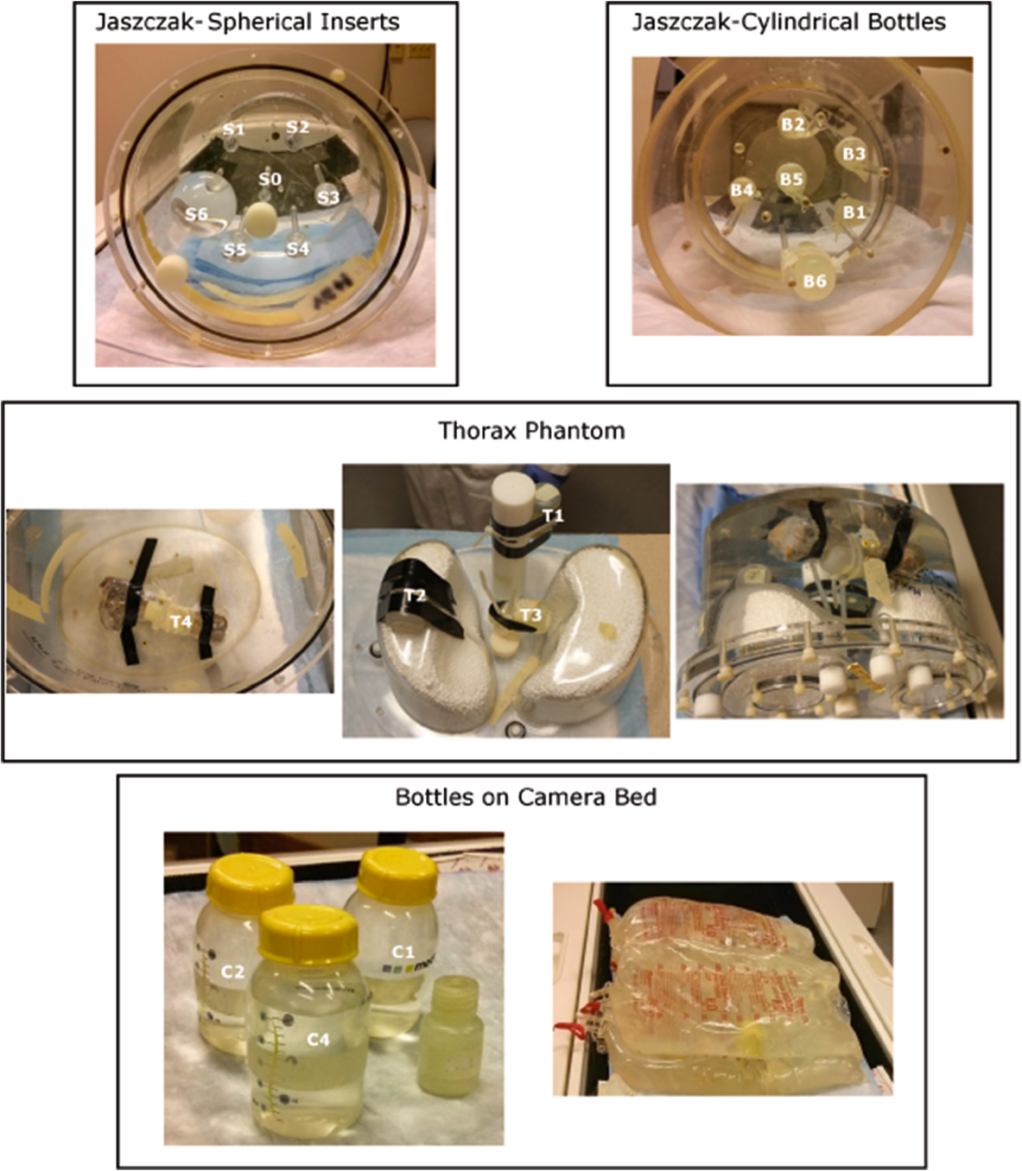
Photos of the phantoms used in the experiments

To test the quantification accuracy of our methods, the first series of experiments used eight different phantom configurations. Photos of the phantoms (described below) are shown in Fig. 1. The volumes and activities of each of the inserts and acquisition times are summarized in Table 2.

**Table 2:** Summary of the phantoms which were used in the experiments performed to test the accuracy of quantification

	Phantom inserts			
Phantom configuration	Projection duration [s]	Name	Volume [ml]	True activity [MBq]
Jaszczak spheres in air	20	S0	0.5	1.7
		S1	1.0	3.2
		S2	2.0	6.1
Jaszczak spheres in cold water	30	S3	4.0	12.3
		S4	8.0	24.5
		S5	16	48.3
Jaszczak spheres in warm water	30	S6	113	351.6
		B1	8.5	42.6
Jaszczak bottles in air	20	B2	8.5	42.2
		B3	12.	61.3
Jaszczak bottles in warm water	20	B4	12	60.0
		B5	16	90.1
		B6	34	90.1
Thorax phantom in cold water	20	T1	34	300.0
		T2	34	302.9
		T3	34	302.9
		T4	34	302.9
Bottles on bed	10	C1	34	—
		C2	63	854.0
Bottles on bed with water bags	10	C3	182	846.5
		C4	199	1182.6

### Jaszczak phantom with spherical inserts

Seven spheres with volumes ranging from 0.5 to 113 ml filled with water containing 3*.*19 ± 0*.*17 MBq/ml of ^177^Lu were placed inside the cylindrical Jaszczak phantom (Data Spectrum Corporation, NC, USA; 22.2 cm diameter, 19.5 cm height). The phantom was first scanned with the spheres in air (referred *to as Jaszczak spheres in air*). Next, the cylinder was filled with water (no activity), (referred to as *Jaszczak spheres in cold water*), and a second scan was performed.

A third scan (referred to as *Jaszczak spheres in warm water*) was performed with activity present in the background. The concentration of activity in the background was equal to 0*.*49 ± 0.03 MBq/ml, resulting in signal-to-background ratio (SBR) of 6*.*4.

### Jaszczak with cylindrical bottle inserts

Next, to evaluate the potential dependence of the accuracy of quantification on the shape of the object, two of the previous scans were repeated with cylindrical inserts. Two 8.5 mL (B1 and B2), two 12 ml (B3 and B4), and one 16 ml (B5) bottles were filled with activity with concentration of 5*.*11 ± 0*.*22 MBq/ml. Similar to spherical inserts study, the phantom was first scanned in air (*Jaszczak: bottles in air)*. A second scan (*Jaszczak: bottles in warm water)* was performed with the phantom filled with water containing concentration of activity of 0*.*104 ± 0*.*003 MBq/ml. The SBR in this case was equal to 49*.*1.

### Thorax phantom

The aim of the third experiment was to determine the accuracy of ^177^Lu quantification in challenging non-uniform attenuation conditions. Four identical bottles (T1–T4) of 34 ml volume were filled with the activity shown in Table 2.

The bottles were attached in different locations inside the thorax phantom (Elliptical Lung-Spine Body Phantom, Data Spectrum Corporation) as follows:

- Bottle T1 was attached to the spine.
- Bottle T2 was placed under one of the lungs.
- Bottle T3 was placed in between the lungs and the spine.
- Bottle T4 was attached to a beef bone placed at the inferior part of the phantom. This bone was used to model a tumor close to an object having true bone attenuation (spine insert only approximates real bone density) and to make the attenuation at the bottom of the phantom less homogeneous.

The phantom was filled with cold water (no activity) and scanned.

### Bottles on the camera bed

The final experimental series aimed to determine the accuracy of quantification in the most difficult case. Four bottles filled with activity were placed between irregularly shaped objects (water bags) creating non-uniform attenuation and scatter conditions. Bottles C1, C2, C3, and C4 were filled with activity (Table 2), and the first scan was performed with bottles placed in air, directly on the camera bed, *bottles on bed*. Then, the bottles were placed between four 2 L water bags (two below and two on top of the bottles) and a second scan was performed, *bottles on bed with water bags*.

### Jaszczak with changing SBR activity concentrations

Besides the eight phantom configurations described above, five additional, separate experiments were performed to determine the calibration curves for our IADT method. In these experiments, six bottles (*V* = 17.0 to 199.5 ml) filled with ^177^Lu activity with concentration of 0*.*62 ± 0*.*03 MBq/ml placed inside a Jaszczak phantom were scanned. Five SPECT/CT scans were performed: for the first scan the phantom was empty so the inserts were scanned in air and for the following four scans, the activity in the large cylinder (background) was gradually increased to obtain four different SBR’s (14*.*0, 8*.*0, 5*.*3, and 4*.*1). The first three of these scans were also used to determine CNF (discussed in the next section) in tomographic mode to be compared with planar scans.

### Planar and tomographic acquisitions for CNF determination

Our third objective was to evaluate the accuracy of different methods that can be used to determine the CNF. To this end, we performed several experiments with ^177^Lu using a MELP collimator. Table 3 summarizes these experiments.

**Table 3:** Details of planar and tomographic acquisitions performed to determine the CNF for ^177^Lu

	Mode (phantom)	Fig. 5 data	Phantom	Source info	Acquisition parameters
A	Planar^a^ (point source)	Planar methods 1 and 2		Activity 11.7 MBq Volume 0.5 ml Scan duration = 10 min	Collimator MELP PW [187.2–228.8] keV LSW [153.0–187.0] keV USW [229.5–280.2] keV
B	Tomographic^b^ (uniformly hot cylinder)	SPECT cylinder		Total activity 659.6 MBq Volume 6500 ml #Projections = 96 Time/projection = 10s	
C	Tomographic (6 bottles in air)	SPECT air		Total activity 233.4 MBq Vol bottles [17–199.5] ml #Projections = 90 Time/projection = 20s	
D	Tomographic (6 spheres in warm water)	SPECT HW1		Total activity 489.1 MBq Vol bottles [17–199.5] ml #Projections = 90 Time/projection = 30s	
E	Tomographic (6 spheres in warm water)	SPECT HW2		Total activity 681.3 MBq Vol bottles [17–199.5] ml #Projections = 90 Time/projection = 30s	

^a^The camera sensitivity does not depend on the source-collimator distance although increase in recorded counts may occur due to septal penetration if the sources are placed very close to the collimator. To minimize this effect, in this study the point sources were placed at 30 cm from the collimator surface

^b^Uniform cylinder was scanned at the L’Hôtel-Dieu de Québec site of the CHU de Québec–Université Laval center (Quebec City, Canada) and dual energy window was performed for scatter correction on this phantom

All scans, except for B (Table 3), were performed at the Nuclear Medicine Department, Vancouver General Hospital (Vancouver, British Colombia) and used three energy windows. The uniform cylinder (scan D) filled with ^177^Lu-chloride was scanned at the *L’Hôtel-Dieu de Québec* site of the *CHU de Québec–Université Laval* center (Quebec City, QC). It used dual energy window (DEW) scatter correction: PW (187.2–228.8 keV) and LSW (166.4–187.2 keV) instead of three windows.

### Image reconstruction

Reconstructions were performed using our in-house developed software incorporated into a graphical user interface (GUI) named single photon emission quantitative tomographic reconstruction (SPEQToR). It allows the user to select the reconstruction algorithm and the corrections to be included in this reconstruction. All reconstructions used the standard ordered subsets expectation maximization (OSEM) algorithm (10 subsets/6 iterations) with CT-based attenuation correction (AC), resolution recovery (RR), and scatter correction (SC).

The number of subsets and iterations applied in the OSEM algorithm were selected based on a small study performed prior to the one described here. Because in patients the sizes of lesions are not known a priori, the chosen number of 10 subsets and 6 iterations seem to provide a good compromise between quantification accuracy for big and small lesions and between accuracy and speed of the reconstruction. Moreover, although it is important to accurately quantify small tumors, kidneys remain the limiting organ in Lu-177 therapies.

Our resolution recovery method incorporates the distance-dependent geometric response of the detector-collimator system (using Gaussian functions) directly into the system matrix. The model does not include septal penetration or scatter. The details of the resolution recovery method are described in Blinder et al. [23].

In imaging studies using ^177^Lu, besides primary photons, the 208 keV photopeak window contains Bremsstrahlung, self-scatter, and high-energy scatter components. Bremsstrahlung for ^177^Lu (^188^Re and ^90^Y) has been already investigated by us and was shown to be negligible (<0.02%) [24]. Although the contribution of high-energy photons also is low in the USW, our simulations indicated that it is not negligible (3%, the number of photons in the PW). Most of the high-energy photons scatter within the crystal of the camera after passing through the collimator. Therefore, better accuracy is expected when these high-energy photons are corrected. In summary, only corrections for the two scatter components have been incorporated into the OSEM formula, but Bremsstrahlung emissions were neglected:

1 $$ {X}_j^{l+1}=\frac{X_j^l}{{\displaystyle {\sum}_{i\in {\Omega}_i}}{C}_{ij}}{\displaystyle \sum_{i\in {\Omega}_i}}{C}_{ij}\ \frac{Y_i}{{\displaystyle {\sum}_k}{C}_{ij}{X}_k^l+S+H} $$

In this equation, *Y*_*i*_ represents the measured projections, *C*_*ij*_ is the system matrix in which attenuation and resolution recovery information are included, and ${X}_j^l$ is the *l*th estimate of the image. The terms *S* and *H* in the denominator represent the self-scatter and high-energy scatter components, respectively.

The two scatter correction methods which were investigated in this study represent the two very different approaches: APDI is an analytical scatter correction method that combines the first estimate of activity distribution in the object with information about its density distribution (attenuation map) with the Klein-Nishina formula to determine *S*, the contribution of scattered photons to the measured projections. Subsequently, the *S* component, together with *H* (the high-energy scatter component which is calculated using counts recorded in USW) are incorporated into the denominator of Eq. (1). The method has been shown to provide accurate quantification for ^99m^Tc [14, 25, 26], ^188^Re [27], and ^177^Lu [13], but is computationally intensive.

TEW, on the other hand, is fast and has been implemented on many SPECT/CT cameras making it widely available in Nuclear Medicine departments. Its further advantage is the fact that counts recorded in LSW and USW provide a joint estimate of *S* + *H* without any need for additional calculations. It is, however, an approximate method that does not model the spatial distribution of scatter [10].

No smoothing was applied to any energy window in either APDI or TEW method.

### Determination of the CNF

For the planar scan (A in Table 3), two methods were tested when determining CNF:

Method 1. Using all counts in the PW, the *CNF* was calculated by dividing the total number of counts in the image (*C*_pw_) by the activity of the source (*A*) and the duration of the scan (*t*_d_).

2 $$ \mathrm{C}\mathrm{N}\mathrm{F}=\frac{C_{\mathrm{pw}}}{A\times {t}_{\mathrm{d}}} $$

Method 2. Using the counts in the PW corrected for scatter with the TEW method, the scattered counts (*C*_s_) were estimated using the counts in the LSW (*C*_ls_), the USW (*C*_us_), and the widths of each window *w*_ls_, *w*_us_, and *w*_pw_ for the LSW, USW, and PW, respectively.

3 $$ {C}_{\mathrm{s}}=\left(\frac{C_{\mathrm{ls}}}{w_{\mathrm{ls}}}+\frac{C_{\mathrm{us}}}{w_{\mathrm{us}}}\right)\frac{w_{\mathrm{pw}}}{2} $$

The primary photons (*C*_prim_) were then determined by subtracting *C*_s_ from counts in the photopeak window *C*_pw_.

4 $$ {C}_{\mathrm{prim}}={C}_{\mathrm{pw}}-{C}_{\mathrm{s}} $$

The CNF was calculated using equation similar to Eq. (2), but with primary photons only.

5 $$ \mathrm{C}\mathrm{N}\mathrm{F}=\frac{C_{\mathrm{prim}}}{\left(A\times {t}_{\mathrm{d}}\right)} $$

For the four tomographic scans (B, C, D, and E in Table 3), the CNF was determined by taking the total number of counts in each reconstructed image (*C*_rec_) and dividing them by the activity in the phantom multiplied by the total scan duration which was given by the number of projections (*n*_p_) multiplied by the duration of each projection (*t*_p_).

6 $$ \mathrm{C}\mathrm{N}\mathrm{F}=\frac{C_{\mathrm{rec}}}{\left(A\times {t}_{\mathrm{p}}\times {n}_{\mathrm{p}}\right)} $$

The reconstructions of the SPECT/CT data were performed using the methods described in section 2.2, with TEW scatter correction.

### Segmentation

All segmentations were performed in three dimensions (3D) and consisted of two steps. First, to separate each of the analyzed objects from other objects in the field of view (FOV), a rough segmentation of the phantom was performed by manually drawing a large region of interest (ROI) around the desired object in each slice. These ROIs were then combined to create a large 3D volume of interest (VOI) around the object which was then used to perform a finer segmentation using one of the methods described below.

For scans in air, a fixed threshold of 0.1% was applied to the data in the large VOI. For scan in cold water, the threshold had to be raised to account for residual scattered counts in the background. For these scans a 1% fixed threshold was applied to the data in the large VOI. The threshold values were determined using the mean of the 9 voxels containing the highest activity values within the initially selected ROIs.

These low-level thresholds were chosen to include counts which due to spill out or partial volume effects are found outside the physical boundary of the object. For scans with warm background, the accuracy of quantification was compared for the three segmentation methods:

1. A fixed 40% threshold.
2. Segmentation based on CT images.
3. Our iterative adaptive dual thresholding (IADT) method [22].

A fixed threshold method is often used for segmentations performed in clinical studies with threshold values usually close to 40% [21]. Therefore, this 40% fixed threshold was used here to serve as a reference for both cases of SBR = 6.4 and SBR = 49.1. Next, for the CT-based segmentation, the volumes of interest were defined by segmenting the CT image, so that for each object, its segmented volume was equal to its physical volume, as shown on the CT.

Finally, the IADT method was used. The IADT method [22] uses two different thresholding curves (one for activity and a different one for volume) to iteratively determine values of the two thresholds which are optimized for the SBR measured in the segmented VOI. There is no need for any a priori information about the target volume or SBR. The calibration curves must be determined in a separate calibration experiment. In our case, acquisitions of the Jaszczak phantom with inserts with four different SBR activity concentrations (section 2.2.5) were used. As described by Grimes et al. [22], the data from these experiments were reconstructed twice, and two separate sets of thresholding calibration curves (one set for TEW and one for APDI) were obtained. These curves were used for the iterative segmentation of the current phantom experiments with a hot background activity.

### Quantification accuracy and statistical analysis

The accuracy of activity quantification in each object was estimated for all phantom configurations, scatter corrections, and segmentation methods. The numerical value of the error of quantification was calculated as the percentage difference of the object activity determined using each of the analyzed methods relative to the “true” activity as measured in the dose calibrator. According to the dose calibrator manufacturer (Capintec), the variability of these measurements is equal to 5%. Box plots were generated for each phantom configuration and for both scatter correction methods.

A statistical analysis of the errors for images reconstructed using TEW and APDI scatter corrections methods was performed. As the distributions of these errors showed to be non-Gaussian, the Mann-Whitney test was applied to check the null hypothesis that the two scatter correction methods provide the same accuracy of activity quantification (the hypothesis was accepted for *p* < 0.05). This was done separately for each phantom configuration, and in the case of hot water for the three segmentation methods.

Additionally, to compare the three segmentation methods used in the warm water phantom configurations, a Mood’s median test was applied. This test allows for the comparison of the three segmentation methods at once. In this case, the null hypothesis assumed that the segmentation methods provided the same results (the hypothesis was accepted for *p* < 0.05).

## Results

### Phantom experiments

**Fig. 2:**
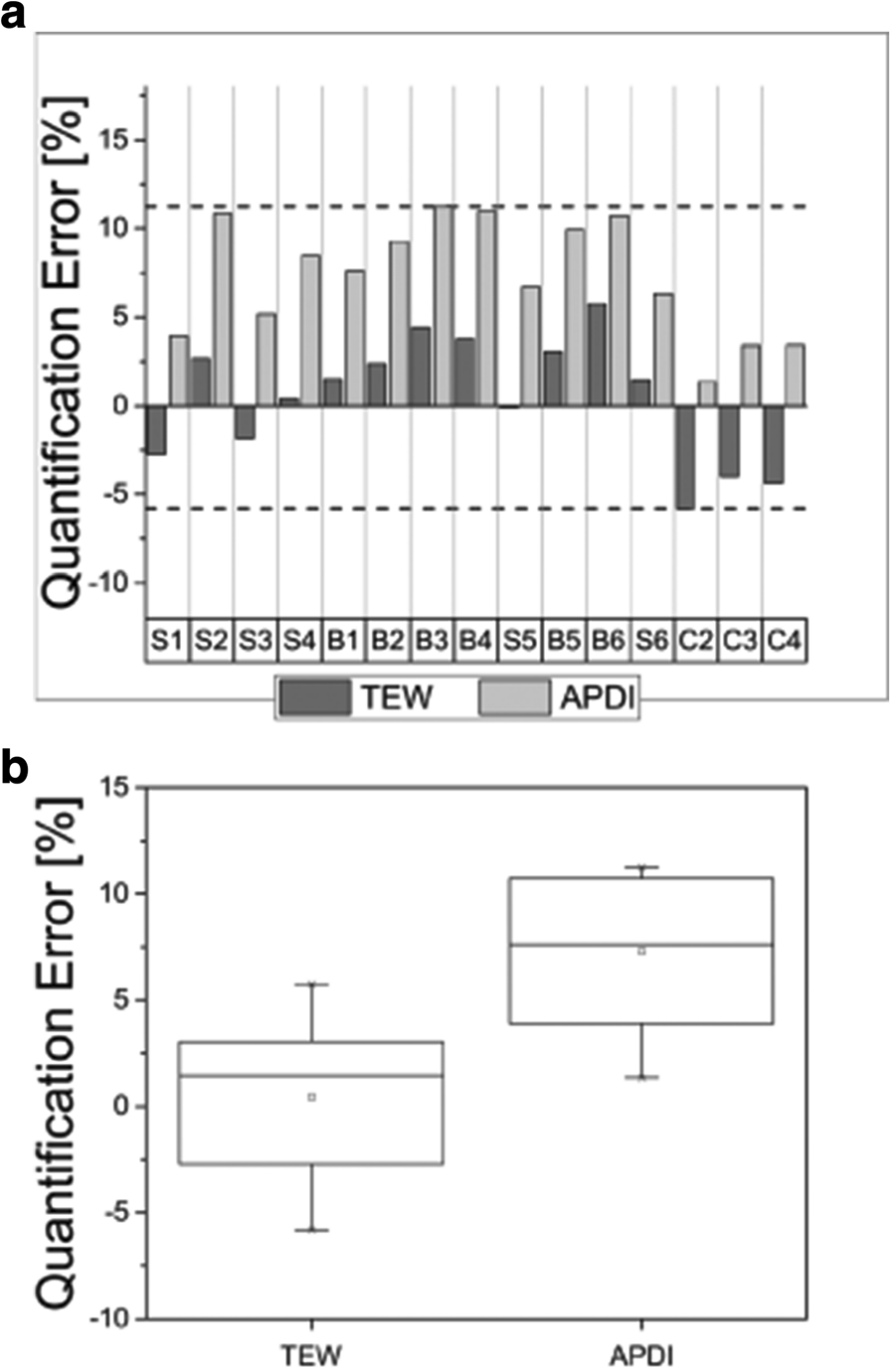
Quantification errors for phantom inserts with different shapes and volumes scanned in air (**a**), and error distribution for both scatter correction methods, TEW and APDI (**b**). The *horizontal dashed lines* in (**a**) mark the range (maximum and minimum) of the deviations from the truth. The *boxes* in (**b**) represent the range of variation (interquartile range-IQR) of the distributions

**Fig. 3:**
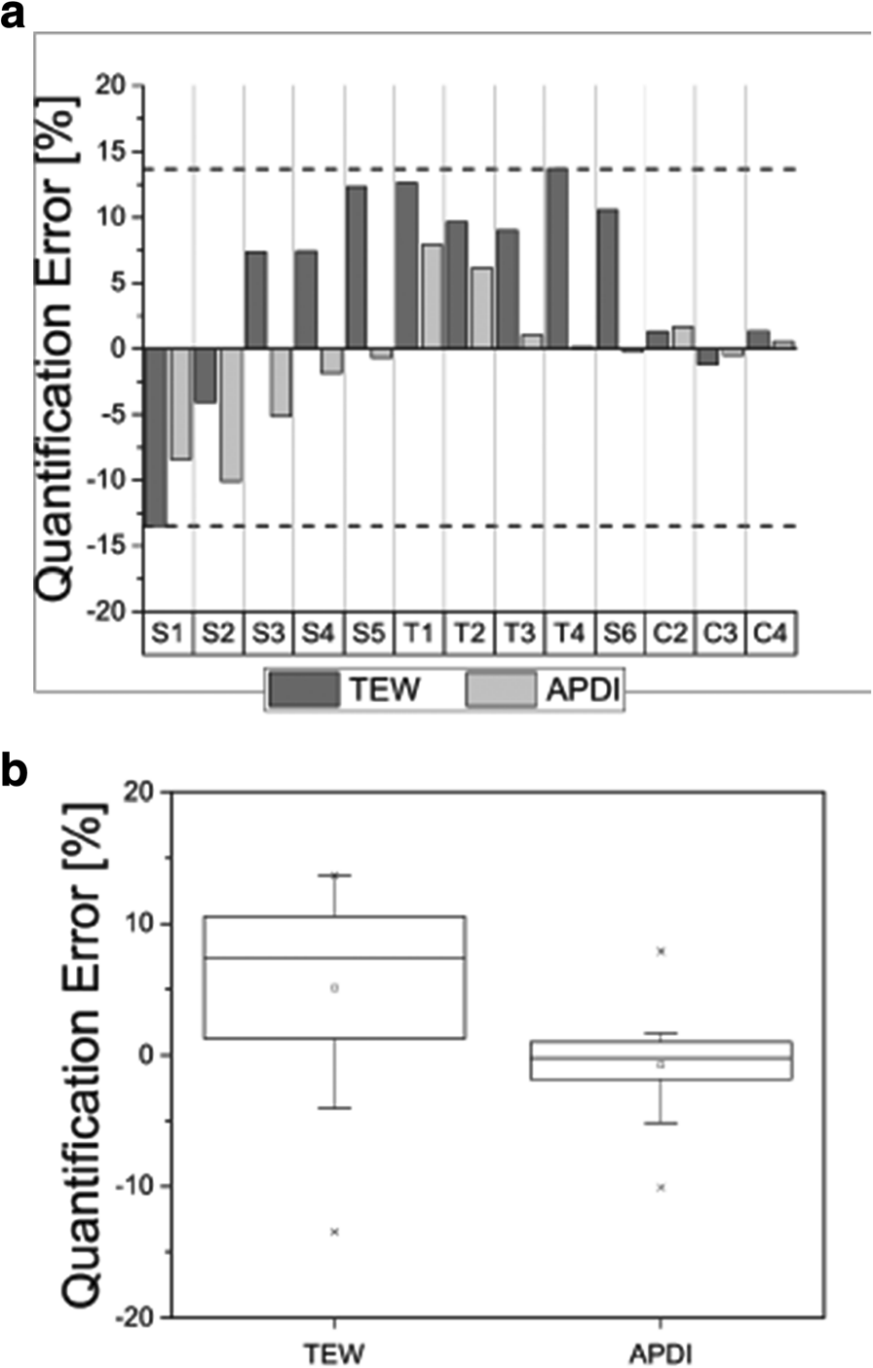
Quantification errors for phantom inserts with different shapes and volumes scanned in cold water and error distribution (**b**) for both scatter correction methods, TEW and APDI (**b**). The *horizontal dashed lines* in (**a**) mark the maximum and minimum deviations from the truth. The *boxes* in (**b**) represent the range of variation (interquartile range-IQR) of the distributions

**Fig. 4:**
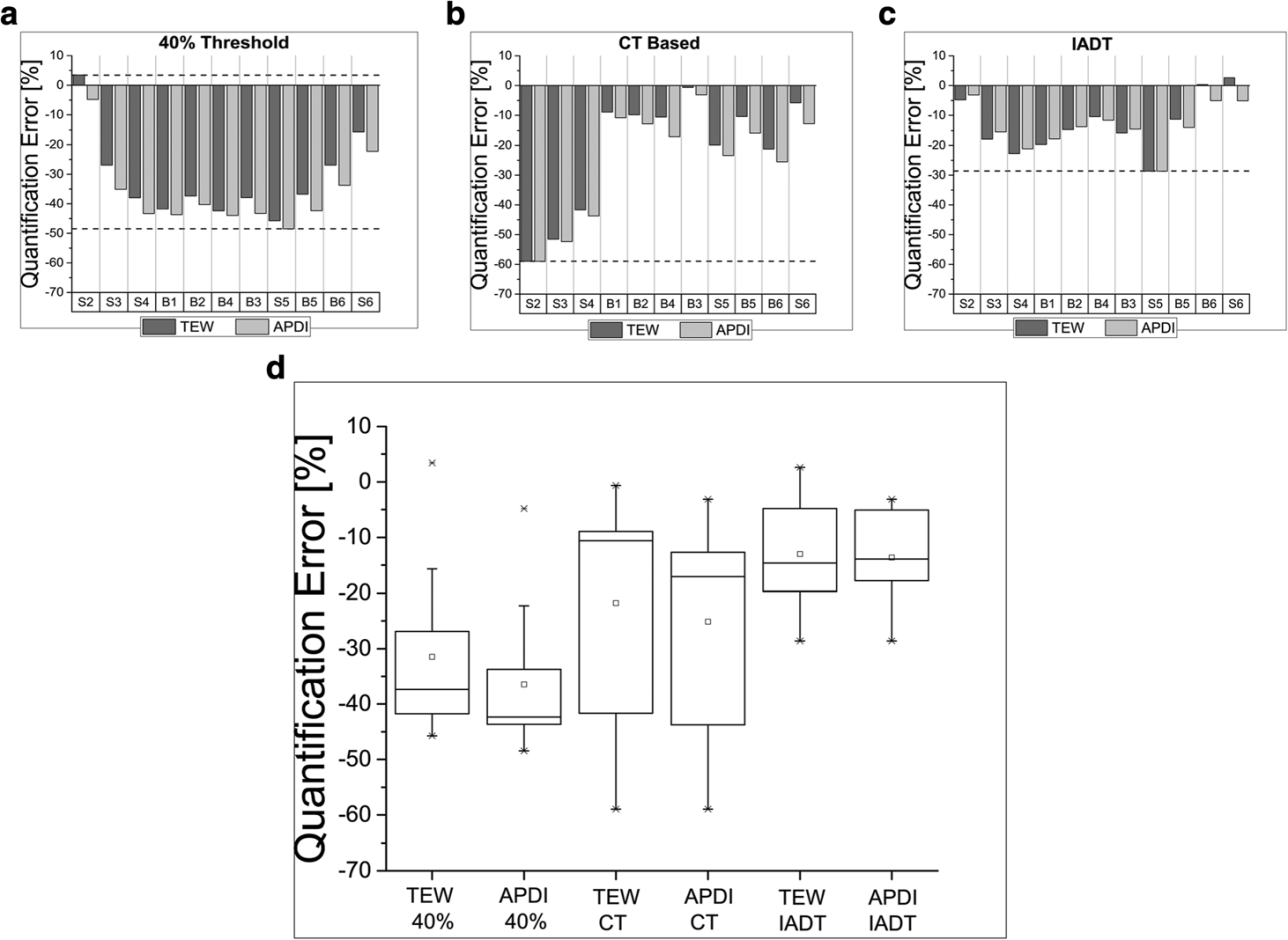
Quantification errors (with uncertainties) for phantom inserts with different shapes and volumes scanned in warm water and segmented with three different methods: 40% fixed threshold (**a**), CT based (**b**), and IADT (**c**). The error distribution for both scatter correction methods, TEW and APDI (**d**). The *horizontal lines* in (**a**–**c**) mark the maximum and minimum deviations from the truth. The *boxes* in (**d**) represent the range of variation (interquartile range-IQR) of the distributions

Figures 2, 3, and 4 summarize the results of the analysis of activity quantification in objects of different sizes and shapes placed in air, cold water, and warm water, respectively. In the top part of the figures, the quantification errors for individual objects, sorted by increasing volumes from the left (S1) to the right (C4), are presented. The distributions of accuracy errors for both scatter corrections and, in case of scans in warm water—for the three segmentation methods are shown in the lower part of each figure. In all cases, the CNF values obtained using the planar method 2 was used. Please note that the following objects were not included in our analysis: (a) sphere S0 was excluded due to its very small size, (b) bottle C1 was not analyzed because we discovered that a large error was made in determination of its activity, and (c) sphere S1 was not analyzed because it was not visible in images reconstructed from scans with hot water.

In general, for phantoms scanned in air and reconstructed with APDI scatter correction method, the activities were overestimated by up to 11%, while TEW results were closer to the true values of activity. For scans performed in cold water, the activity in volumes smaller or equal to 2 ml was underestimated by both scatter correction methods, while for larger volume reconstructions with APDI outperformed those done with TEW. This effect was especially noticeable for bottles scanned in the non-uniform thorax phantom. In particular, bottle T4, which was attached to the bone, showed excellent quantification accuracy for APDI. Finally, for the large bottles (160–200 ml) placed on the camera bed and surrounded by the water bags, both scatter correction methods behaved very similarly, providing very good accuracy of activity quantification.

### Determination of the CNF

**Fig. 5:**
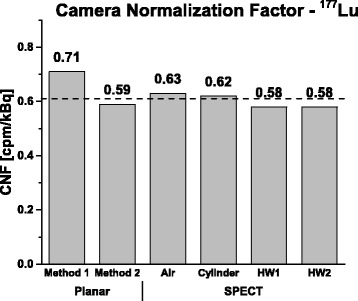
Camera normalization factors (CNF) ^177^Lu obtained using different methods. The *horizontal dashed line* represents the average value as determined from the planar method 2 and the tomographic acquisitions

Figure 5 shows the normalization factors obtained with both planar and tomographic scans of ^177^Lu sources. The values obtained from all tomographic acquisitions agree within 7% with each other and with the values obtained from planar scan, providing that the TEW scatter correction was applied (i.e., method 2).

### Segmentation

Figures 4a, b, and c compare the accuracy of quantification achieved with the three segmentation methods for objects scanned in the hot background. The objects have been sorted by volumes from the smallest object (S2) to the largest one (S6). Both 40% fixed threshold and CT-based segmentation methods grossly underestimated the activity for both TEW and APDI scatter correction methods. The IADT segmentation method seemed to be the most effective in recovering the true activity of each object. For small volumes (<17 ml), due to spill out caused by partial volume effects (PVE), the activity estimates were not very accurate (>10%). For objects larger than 34 ml errors below, 10% were achieved.

## Discussion

### ^177^Lu quantification

For objects placed in air (Fig. 2), the effects of attenuation and scatter were small and mostly due to attenuation and scatter within the object itself. Additionally, segmentation using fixed threshold with a very low value of 0.1% allowed us to recover the majority of activity in each object. Therefore, in general, the accuracy of activity quantification achieved in these experiments was very good; activities reconstructed with TEW scatter correction differed for most of the objects by less than 5% from the true values. These results confirmed that our reconstruction method perform well in low scatter and attenuation conditions.

It is interesting to note that for scans in air, the results obtained from the reconstructions with TEW were better than those from the APDI method, which in all cases overestimated the activities. This effect, especially strong for smaller objects, can be explained by the fact that although APDI accurately models scatter distributions, it calculates scatter using attenuation maps of the object with large (interpolated) voxels. Large PVE at the boundary between water and air caused APDI method to underestimate scatter contributions.

On the other hand, when bottles were placed in air directly on the camera bed (C2, C3, and C4), although the results from APDI still overestimated the activity, its accuracy was better than TEW, with errors lower than 3%. In this case, the volumes were larger, so PVE was relatively small and non-uniform scatter conditions (due to close proximity of the camera bed) were modeled better by APDI than by the TEW method.

For scans performed with cold water background (no activity), the reconstructions with TEW scatter correction underestimated the activity in small objects and overestimated it in large objects. Furthermore, the reconstructions with APDI provided more accurate activity values and also qualitatively sharper images than those reconstructed with TEW. In the thorax phantom (non-uniform attenuation case), both scatter correction methods overestimated the activity of the inserts (T1, T2, T3, and T4), but again, APDI was more or even much more accurate than TEW. In particular, activities in bottle T3 (which was placed between the lungs and spine) and in bottle T4 (which was placed in contact with the beef bone) were both highly overestimated by TEW while APDI reconstructed the true activity very accurately (error <1%). Finally, for the three large bottles placed between water bags (C2, C3, and C4), both scatter correction methods performed equally well.

Figures 2 and 3 do not show a big difference between quantification errors of small objects and big objects. Usually when fixed threshold values are chosen, the effect of partial volume effects is larger for small objects making their activity quantification worse than for large objects. However, the fixed thresholds used in this study made the partial volume effects negligible.

Figures 2b, 3b, and 4d show box plots summarizing quantification errors for different phantom scans. These plots are especially useful when comparing the performance of APDI and TEW scatter correction methods. In clinics, lesions of different sizes and locations might be present in the same patient and quantification accuracy in these cases will vary. Therefore, it is important to see the range of these variations and dispersion of the accuracy which can be achieved with the two scatter correction methods, to identify potential advantages of each method. Smaller variations around the mean suggest a better overall performance as long as quantification remains within a few percent from the truth.

For the scans in air (Fig. 2b) and cold water (Fig. 3b), the Mann-Whitney test showed that distributions of TEW and APDI were significantly different. In particular, for the scans in air, the mean quantification error in images reconstructed with TEW was close to 1% of the true value. However, the distribution of errors was skewed towards negative values, meaning that TEW was likely to underestimate the activity. This effect was especially pronounced in scans with large bottles (C1, C2, and C3) placed directly on the camera bed, as TEW performed better when the objects are further from the scattering medium. As discussed before, for the scans in air, the reconstructions with APDI in general overestimated the activity values, but produced more accurate results in a non-uniform attenuation environment.

For the scans in cold water, the box plot (Fig. 3b) shows that the distributions of errors for both TEW and APDI were skewed towards lower values (the mean is lower than the line representing the median). While reconstructions with TEW tended to overestimate the activity, those performed with APDI mostly underestimated it. On average, the APDI results were more accurate than TEW and the error variation about the mean value was smaller.

The situation became more challenging when warm background was present. In this case, sphere S1 had been removed from the analysis because, due to its small volume (1 ml), it could not be distinguished from the background. For the same reason, the quantification results for sphere S2 (2 ml), although reported here, may not be trusted.

For the scans in warm water (Fig. 4d), the Mann-Whitney test did not show statistically significant differences between the two scatter correction methods when data were analyzed using the same segmentation algorithm.

Our analysis of the three segmentation methods showed that activity in the objects determined using the 40% threshold, which is often used in clinical studies [21], was grossly underestimated. This threshold value clearly was too high and did not properly account for the spill out effect. Segmentations which used the true dimensions of each object (based on CT images) were strongly affected by PVEs, especially for the smallest spheres (S2–S4). The accuracy obtained for small spheres was similar to that achieved with the 40% threshold (errors ~40–60%), but for larger objects, the CT-based method performed substantially better than the 40% threshold. Interestingly, results were better for small cylindrical objects (B1 and B2 bottles, 8.5 ml volume, errors <15%)) than for the spheres having approximately the same volumes (S4 sphere, 8 ml volume, error ~40%). However, the CT-based segmentation method may be difficult to use in patient studies because boundaries of organs/tumors are not always visible in CT images. One way to deal with this problem is to apply recovery coefficients, as was done in the study by Ilan et al. [28]. They scanned a known phantom and used a 42% fixed threshold for segmentation. Then, for each object size, they calculated recovery coefficient to correct for missing activity.

In general, the best results were obtained with the IADT segmentation method. In particular, for volumes equal or larger than 34 ml, the accuracy of quantification was better than 5%. Good performance of the IADT method is at least partly due its “adaptive” character, i.e., the thresholding curves that were used in segmentation have been obtained from phantom scans reconstructed with the same method (TEW and APDI) as the images which were segmented. Please note, however, that the IADT method fails when activity distribution in the analyzed VOI is non-uniform as then the SBR cannot be correctly determined (see [22]). Therefore, the quality of each object’s segmentation must be critically evaluated by the operator.

Finally, the box plot of Fig. 4d summarizes the results for the three segmentation and two scatter correction methods. Because all experiments with warm background involved phantoms with uniform distribution of attenuating medium, the quantification accuracy of both scatter correction methods was very similar for all three segmentation approaches. Nevertheless, all three segmentation methods underestimate the activity indicating the need for improvement in segmentation techniques. The Mood’s median test showed that the three methods (i.e., 40%, CT, and IADT segmentations) were significantly different for the samples reconstructed using the two scatter correction methods.

Additionally, if imaged activities are very high, the count losses caused by camera dead time must be corrected for. In our phantom experiments the activities were relatively low, so no dead time corrections were necessary. Nevertheless, this topic has been extensively investigated by us in a separate study and will be reported shortly.

### Camera normalization

Although methods to determine camera normalization factor have been extensively investigated, there is no consensus which approach is the best. Dewaraja et al. [29] used Monte Carlo simulations of ^131^I activity to compare two approaches: a planar scan of a point source and two tomographic scans of a phantom uniformly filled with activity and the same phantom filled with water with a hot sphere placed at the center. They found that the camera normalization obtained using hot sphere placed inside the warm-water-filled phantom provided better quantification than the uniform phantom. However, if sufficiently large volume of interest was used, accuracies of quantification obtained from the point source and water cylinder were similar. Ljungberg et al. [30] compared simulations and experimentally measured CNF using a planar scans of the ^131^I point source. When counts in the whole field of view (FOV) of the camera were taken into account, good agreement was obtained between simulations and measurement. Zeintl et al. [31] used a cylinder uniformly filled with ^99m^Tc and reported CNF values similar to those obtained by our group using planar scans of point sources [26]. Frey et al. [32] suggests that the use of a phantom tomographic acquisition, instead of a planar scan of a small source, is the optimal approach. Lastly, D’Arienzo et al. [33] performed tomographic scans of point sources in air and extended phantoms and showed better quantification accuracy for CNF determined from phantom studies than from point sources. They attribute this result to the fact that attenuation and scatter corrections were not incorporated into the reconstruction of the point source.

The results of our CNF determination are presented in Fig. 5. In general, the values of normalization determined using the planar method 2 (with scatter correction) and all tomographic scans were very similar. However, when scatter correction was not applied (method 1), the CNF values were overestimated.

Also*,* the CNF values obtained from the planar method 2 and the tomographic scans agree well, and the maximum deviation from the mean remains below 5%. Due to contribution from scatter of high-energy photons, planar method 1 produced higher CNF value than the other methods.

Although our experiments were performed on a Siemens SymbiaT camera, our experience indicates that similar results should be expected for data collected using other modern cameras. It is important, however, that CNF is correctly determined. Our results suggest that planar scans of point sources placed in air are sufficient for the determination of the camera calibration factor. However, for planar scans, scatter correction should be applied to remove contributions from background and scattered photons. This is particularly important for ^177^Lu and other isotopes in which background from high energy scattered photons can be substantial.

## Conclusions

The accuracy of activity determination was evaluated for ^177^Lu imaging studies performed according to MIRD Pamphlet 23 and 26 recommendations. Several phantoms containing inserts with a variety of shapes and sizes placed in different attenuation conditions were performed.

Our results showed that for phantoms scanned in air, errors in activity quantification below 6.5 and 11.5% were achieved for large (i.e., >100 ml) and small (i.e., <100 ml) objects, respectively, for both TEW and APDI scatter correction methods. For phantoms scanned in cold water, APDI provided activities within 2% of the true values for volumes >100 ml, and within 10% for smaller objects. While APDI performed better in challenging situations involving non-uniform attenuating medium, for scans of phantoms with uniform density, the performance of both scatter correction methods was very similar, indicating that much faster TEW method can be sufficient when scanning body areas with relatively uniform tissue distributions.

For scans with activity added to the phantom’s background, all three segmentation methods (40% fixed threshold, CT-based, and our IADT method) underestimated the activity in the inserts. However, our quantification results for scans in air and cold water suggest that our modeling of scatter and attenuation are sufficiently accurate. However, segmentation is still a challenge. For both TEW and APDI scatter correction methods, IADT segmentation provided the best quantification accuracy. For IADT, the fact that only small differences were seen between the two scatter correction algorithms could be attributed to the fact that the threshold curves which were used to segment objects were created using reconstructions with the same sets of corrections as those used in the phantom reconstructions.

Regarding the camera calibration, our results showed that, when TEW corrections are applied, the CNF values determined using planar scans of the point sources were the same as those from the tomographic scans of extended sources. This is an important finding, considering that planar scans of point sources are much easier to perform than tomographic acquisitions of extended phantoms.

In summary, although the exact activity quantification (thus also dose calculation) of very small lesions still remains challenging, IADT segmentation performed well, within 5%, for larger inserts (>34 ml), thus it would be appropriate for use in dosimetry calculation of medium to large organs, like the kidneys. Finally, for lesions located in regions with non-uniform attenuation, the APDI scatter correction method would be recommended.

The results of this work strongly suggest that in patient scans, similarly good quantification accuracy can be achieved in determination of activity in larger organs and/or tumors. This is an important conclusion, as the exact quantification of activity in an organ is crucial for the accuracy of dosimetry calculation for this organ and such dosimetry is particularly important for critical organs, such as, for example, the kidneys.

Our results indicate that the methods used in this study allow for accurate determination of ^177^Lu activity, but also suggest that organ/tumor segmentation still remains challenging. Further efforts should be made in developing better segmentation algorithms for nuclear medicine data.

## Additional file

Additional file 1: Table S1. Quantification error data for Figs. 2 and 3, inserts in air and water. **Table S2.** Quantification error data for Fig. 4, inserts in hot water. (DOC 67 kb)

## Abbreviations

AC: Attenuation correction
APDI: Analytical photon distribution interpolated
CNF: Camera normalization factor
DEW: Double energy window
FOV: Field of view
IADT: Interative adaptive dual thresholding
LEHR: Low energy high resolution
LSW: Lower scatter window
MELP: Medium energy low penetration
MIRD: Committee on medical internal radiation dose
NETs: Neuroendocrine tumors
OSEM: Ordered subsets expectation maximization
PVE: Partial volume effects
PW: Photopeak window
ROI: Region of interest
RR: Resolution recovery
SBR: Signal-to-background ratio
SC: Scatter correction
TEW: Triple energy window
TRT: Targeted radionuclide therapy
USW: Upper scatter window
VOI: Volume of interest
