# Supplementary material for: Accuracy of 177Lu activity quantification in SPECT imaging: a phantom study
Source: EJNMMI Phys. 2017 Jan 7;4:2. doi: 10.1186/s40658-016-0170-3 (PMC5218957; doi:10.1186/s40658-016-0170-3)
Supplement: Additional file 1: Table S1. — Quantification error data for Figs. 2 and 3, inserts in air and water. Table S2. Quantification error data for Fig. 4, inserts in hot water. (DOC 67 kb) [file 40658_2016_170_MOESM1_ESM.doc]

Table S1: Quantification Error data for figures 2 and 3, inserts in air and water.

| **Medium** | **Insert** | **Volume** | **TEW Quantification Error** | **APDI Quantification Error** |
| --- | --- | --- | --- | --- |
| Air | S1 | 1.0 | -2.7 | 3.9 |
|  | S2 | 2.0 | 2.7 | 10.8 |
|  | S3 | 4.0 | -1.8 | 5.2 |
|  | S4 | 8.0 | 0.4 | 8.5 |
|  | B1 | 8.5 | 1.5 | 7.6 |
|  | B2 | 8.5 | 2.4 | 9.3 |
|  | B3 | 11.7 | 4.4 | 11.3 |
|  | B4 | 11.7 | 3.8 | 11.0 |
|  | S5 | 16.0 | -0.1 | 6.7 |
|  | B5 | 16.2 | 3.1 | 9.9 |
|  | B6 | 34.0 | 5.7 | 10.7 |
|  | S6 | 113.1 | 1.5 | 6.3 |
|  | C2 | 163.1 | -5.8 | 1.3 |
|  | C3 | 182.1 | -4.0 | 3.4 |
|  | C4 | 199.1 | -4.4 | 3.4 |
| Water | S1 | 1.0 | -13.5 | -8.4 |
|  | S2 | 2.0 | -4.0 | -10.1 |
|  | S3 | 4.0 | 7.4 | -5.1 |
|  | S4 | 8.0 | 7.4 | -1.8 |
|  | S5 | 16.0 | 12.3 | -0.7 |
|  | T1 | 33.3 | 12.6 | 7.9 |
|  | T2 | 33.5 | 9.7 | 6.2 |
|  | T3 | 33.8 | 9.0 | 1.0 |
|  | T4 | 34.2 | 13.7 | 0.2 |
|  | S6 | 113.1 | 10.5 | -0.2 |
|  | C2 | 163.1 | 1.3 | 1.7 |
|  | C3 | 182.1 | -1.2 | -0.5 |
|  | C4 | 199.1 | 1.3 | 0.5 |

Table S2: Quantification Error data for figure 4, inserts in hot water.

|  |  | **40%** | | **CT** | | **IADT** | |
| --- | --- | --- | --- | --- | --- | --- | --- |
| **Insert** | **Volume** | **TEW Quantification Error** | **APDI Quantification Error** | **TEW Quantification Error** | **APDI Quantification Error** | **TEW Quantification Error** | **APDI Quantification Error** |
| S2 | 2.0 | 3.4 | -4.8 | -59.0 | -58.9 | -4.8 | -3.1 |
| S3 | 4.0 | -26.7 | -35.1 | -51.6 | -52.4 | -17.9 | -15.4 |
| S4 | 8.0 | -38.0 | -43.3 | -41.7 | -43.7 | -22.8 | -21.1 |
| B1 | 8.5 | -41.8 | -43.7 | -8.9 | -10.8 | -19.7 | -17.8 |
| B2 | 8.5 | -37.4 | -40.3 | -9.8 | -12.7 | -14.7 | -13.7 |
| B4 | 11.7 | -42.4 | -43.9 | -10.6 | -17.1 | -10.4 | -11.6 |
| B3 | 11.7 | -37.9 | -43.3 | -0.6 | -3.1 | -15.8 | -14.5 |
| S5 | 16.0 | -45.7 | -48.5 | -19.9 | -23.4 | -28.6 | -28.6 |
| B5 | 16.2 | -36.8 | -42.3 | -10.4 | -15.8 | -11.3 | -13.9 |
| B6 | 34.0 | -26.9 | -33.7 | -21.2 | -25.5 | 0.3 | -5.1 |
| S6 | 113.1 | -15.7 | -22.3 | -5.8 | -12.7 | 2.6 | -5.1 |
